# Prostanoid Receptors in the Human Vascular Wall

**DOI:** 10.1100/tsw.2007.184

**Published:** 2007-09-01

**Authors:** Xavier Norel

**Affiliations:** INSERM U698: Haemostasis, Bioengineering and Cardiovascular Remodeling CHU X. Bichat, Secteur C. Bernard, 46 rue Henri Huchard, 75877 Paris Cedex 18, France

**Keywords:** prostaglandin, prostacyclin, thromboxane, endothelium, smooth muscle, vasoconstriction, vasodilatation, leukocyte, angiogenesis, migration, proliferation, atherosclerosis, hypertension, aneurysm, PGI_2_, PGE_2_, EP_1_, EP_2_, EP_3_, EP_4_, PPAR, CRTH2, COX-1, COX-2

## Abstract

The mechanisms involved in vascular homeostasis and disease are mostly dependent on the interactions between blood, vascular smooth muscle, and endothelial cells. There is an accumulation of evidence for the involvement of prostanoids, the arachidonic acid metabolites derived from the cyclooxygenase enzymatic pathway, in physiological and/or pathophysiological conditions. In humans, the prostanoids activate different receptors. The classical prostanoid receptors (DP, EP_1–4_, FP, IP, and TP) are localized at the cell plasma or nuclear membrane. In addition, CRTH2 and the nuclear PPAR receptors are two other targets for prostanoids, namely, prostacyclin (PGI_2_) or the natural derivatives of prostaglandin D_2_. While there is little information on the role of CRTH2, there are many reports on PPAR activation and the consecutive expression of genes involved in the human vascular system. The role of the classical prostanoid receptors stimulated by PGI_2_ and thromboxane in the control of the vascular tone has been largely documented, whereas the other receptor subtypes have been overlooked. There is now increasing evidence that suggests a role of PGE_2_ and the EP receptor subtypes in the control of the human vascular tone and remodeling of the vascular wall. These receptors are also present on leukocytes and platelets, and they are implicated in most of the inflammatory processes within the vascular wall. Consequently, the EP receptor subtypes or isoforms would provide a novel and specific cardiovascular therapeutic approach in the near future.

Prostanoids (prostaglandin [PG] and thromboxane [Tx]) are derived from membrane phospholipids and the metabolism of arachidonic acid via the rate-limiting enzyme prostaglandin H synthase more commonly known as cyclooxygenase (COX). After the initial synthesis of $PGH_2$, the production of the other prostanoids will depend on the different respective prostanoid synthase, such as PGE-synthase (PGES) for $PGE_2$. The synthesis of prostanoids implicated in vascular homeostasis is dependent on the presence or absence of each enzymatic activity in the different cells of the blood or the vascular wall.

The different effects of prostanoids are also dependent on the activation of specific receptors, namely, the eight classical prostanoid receptors (DP, $EP_{1–4}$, FP, IP, and TP; Table 1), as well as the recently described chemoattractant receptor CRTH2 and nuclear receptors (PPARα,δ,γ). There is a considerable amount of information on the prostanoid receptors in mice and this will be treated in paper Matsuoka et al[162]. The present review will focus only on the human prostanoid receptors. In the nomenclature for the classical prostanoid receptors, the first letter indicates the prostanoid with the greatest affinity for this receptor. In addition, some isoforms derived from splice variants have been described in human tissues for the $EP_3$ receptor ($EP_{3-I}$, $EP_{3-II}$, $EP_{3-III}$, $EP_{3-IV}$, $EP_{3-V}$, $EP_{3-VI}$, $EP_{3-e}$, $EP_{3-f}$)[1], the FP receptor (FP-A, FP-B), and the TP receptor (TP-α, TP-β)[2]. These receptors, as well as the CRTH2, are found on the cytoplasmic membranes and they are seven transmembrane domain G-protein coupled receptors (Table 1). Their activation leads to increased or decreased production of different intracellular second messengers (Table 1). This situation has been further complicated by the increasing evidence for the presence of the classical prostanoid receptors at the nuclear and/or perinuclear region[3]. The prostanoid receptor localized on the plasma membrane elicits immediate physiological actions, whereas the nuclear one conveys gene regulation. Finally, in order to characterize these different prostanoid receptors, specific pharmacological tools (Table 1) and primers for molecular biology have been described[4,5,6].

**TABLE 1**
**Types and Subtypes of Classical Prostanoid Receptors***

| Prostanoid Receptor (Swiss-Prot n°) | Cloning Ref. | G-Protein Coupled | Second Messengers | Synthetic Agonist | Synthetic Antagonist |
|---|---|---|---|---|---|
| DP (Q13258) | [7] | Gs | cAMP ↑ | BW245C; L644698 | BWA848C; AH6809; ONO-AE3-237 |
| $EP_1$ (P34995) | [8] | Unknown | $Ca^{2+}$ ↑ | 17-Ph-$PGE_2$; Iloprost; Sulprostone | AH6809; SC-19220; SC-51322; ONO-8713 |
| $EP_2$ (P43116) | [9] | Gs | cAMP ↑ | Butaprost; AH13205; ONO-AE1-259; Misoprostol | AH6809 |
| $EP_3$ (P43115) | [1] | Gi (Gs, Gq) | cAMP ↓ (cAMP ↑, IP3 ↑) | ONO-AE248; SC-46275; GR-63799; M&B-28767; Sulprostone; Misoprostol | L826266 |
| $EP_4$ (P35408) | [10] | Gs | cAMP ↑ | ONO-AE1-329; L-902688; Cicaprost | EP4A; AH23848; AH22921; GW627368X |
| FP (P43088) | [11] | Gq | IP3 ↑ | Latanoprost; Fluprostenol; Cloprostenol | |
| IP (P43119) | [12] | Gs (Gq) | cAMP ↑ (IP3 ↑) | Cicaprost; Iloprost; Beraprost | RO1138452 |
| TP (P21731) | [13] | Gq (Gi, Gs) | IP3 ↑ (cAMP ↓ ↑) | U46619; $STA_2$ | BAY u3405; GR32191; AH23848 |

* Molecular and pharmacological characteristics of human prostanoid receptors derived from Swiss-Prot (http://www.expasy.org/sprot/) and IUPHAR (http://www.iuphar-db.org/iuphar-rd/index.html) web sites. The G-proteins and their respective second messengers are indicated in parenthesis when they are not the major one associated with this receptor[14,15].

## PROSTANOID PRODUCTION IN THE HUMAN VASCULAR WALL

The cell or tissue production of prostanoids is dependent on physiological conditions. In normal physiological conditions, prostanoid synthesis is dependent on the COX-1 activity, the constitutive enzyme isoform. The synthesis and release occurs a few minutes after cell or tissue stimulation. In vascular preparations submitted to inflammatory conditions, hypoxia, shear stress, or mechanical perturbation (gravity, stretching) for several hours, the expression of COX-2 and other prostanoid synthase isoforms can be observed.

In normal physiological conditions, the release of $PGI_2$ quantified by the measurement of its stable metabolite (6-keto-$PGF_{1\alpha}$) has been largely described in human endothelial cells in culture after stimulation with thrombin, histamine, adenosine nucleotides (ADP, ATP), and bradykinin[16,17]. In fresh isolated vascular preparations, acetylcholine, serotonin, or leukotrienes induced endothelium-dependent relaxations[18,19,20,21]. These responses were partially inhibited by a nonselective COX inhibitor (indomethacin) and they are dependent on $PGI_2$ release by the endothelium. In these vascular cells or tissue, the production of $PGI_2$ was two- to eightfold greater in comparison with the other prostanoids synthesized[22,23]. These measurements are dependent on the initial COX-1 activity and not the COX-2 activity, since immunohistochemistry experiments and/or western-blot analysis have shown a preferential presence of the COX-1 in the endothelium of ovarian, pulmonary, or aortic vessels [24,25,26,27].

In the human endothelial cells in culture submitted to hypergravity during a period of 24–48 h, the induced expression of COX-2 was observed in association with an increased production of $PGI_2$[28]. Similar effects are observed with the human umbilical vein endothelial cells (HUVEC) under shear stress conditions[29]. Under hypoxia (2 h), the synthesis and the release of $PGF_{2\alpha}$ are induced in human coronary endothelial cells, human microvascular endothelial cells (HMEC), and HUVEC in culture[30]. In addition, cytokines (interleukin [IL]-1$\alpha$/$\beta$; tumor necrosis factor [TNF]-$\alpha$), angiotensin II (Ang II), arginine-vasopressine (AVP), certain growth factors (epidermal growth factor [EGF], transforming growth factor [TGF]-$\alpha$), and endothelin-1 (ET-1) induce the synthesis and release of $PGI_2$ and $PGE_2$ in the majority of the human endothelial cells in culture[31,32,33,34]. These productions are detectable after a few hours of stimulation, implying the induction of the COX-2 isoform in the majority of the cases[31,35,36]. Furthermore, one of the PGES isoforms, the microsomal PGES-1 (mPGES-1), has received much attention because this enzyme is inducible and functionally linked with COX-2 expression. The presence of the transcript coding for the mPGES-1 and the protein after 3 h of HUVEC stimulation with IL-1$\beta$ has been detected[37]. However contradictory results were described[38].

After a few hours of incubation of human vascular smooth muscle cells (HVSMC) with cytokines (IL-1$\beta$, TNF-$\alpha$) and bacterial lipopolysaccharides (LPS), an increased production of $PGI_2$ and $PGE_2$ was also observed in the culture medium[38,39]. Other authors showed that these effects were associated with an induction of the COX-2 isoform[40,41]. For example, the muscular cells of human pulmonary artery in culture (HPASMC) under conditions of hypoxia or in the presence of cytokines express COX-2[25]. HPASMC incubated for 3 h with cytokines and successively stimulated with bradykinin or thrombin for a few minutes have a striking enhanced production of $PGI_2$[42,43]. In these previous studies, $PGE_2$ was not measured. The human fibroblasts are also present in the vascular wall. These cells, after 20 h of CD40 interaction with its ligand express COX-2 and the production of $PGE_2$ by these cells, are increased by a factor 10[44]. In the HVSMC and the human fibroblasts in culture, PGES transcript is present and its expression is increased in the presence of cytokines[38].

As an illustration of the previous experimental results on cell cultures, there is an increased presence of COX-2 and the inducible enzyme responsible for $PGE_2$ synthesis (mPGES-1) detected by immunocytochemistry in the carotid atherosclerotic plaque of symptomatic patients vs. the asymptomatic ones[45]. In conclusion, in most vascular cells, $PGI_2$ is the major biological active prostanoid produced under normal physiological conditions and when not the case, $PGI_2$ and $PGE_2$ are both equally produced as a consequence of the COX-2 induction.

## CELLULAR LOCALISATION OF CLASSICAL PROSTANOID RECEPTORS

The location of the prostanoid receptors are mainly described in the vascular smooth muscle layer. That is supported by the immunohistochemistry data[46], by the physiological responses of isolated smooth muscle cells[47,48], and by the contraction/relaxation of isolated vascular preparations without endothelium[49,50]. For example, the vasoconstriction of human mammary or pulmonary arteries induced by the TP agonist U46619 was not modified in absence of endothelium[51,52]. On the other hand, there is also some evidence that suggests the presence of prostanoid receptors in human endothelium. The involvement of the different TP isoforms has been described in differential control of HUVEC or renal HMEC migration, however, these results are dependent on the number of passages during cell culture[53,54,55]. In addition, the isolated human pulmonary veins show a greater sensitivity during the contraction induced by U46619 when the endothelium was removed[52]. In a similar way, the relaxation of human hand veins produced after $PGE_2$ or $PGF_{2\alpha}$ stimulation was reduced in the absence of endothelium, whereas those elicited by $PGI_2$ or $PGE_1$ were not modified[56]. These results suggest the presence of endothelial prostanoid receptors and the involvement of an endothelial relaxing factor in these veins. On the contrary, in the human radial artery, an endothelial contractile factor was suggested since a reduced sensitivity to U46619 is measured in absence of endothelium[51]. These endothelial factors could either be released after stimulation of an endothelial prostanoid receptor or by the endothelium in a mechanical way in response to the contraction of the underlying smooth muscle induced by the prostanoids.

## NUCLEAR AND CRTH2 RECEPTORS ACTIVATED BY PROSTANOIDS

There are two kinds of vascular nuclear prostanoid receptors: the peroxisome proliferator activated receptor (PPAR) and the classical prostanoid receptors. The location and the role of these classical prostanoid receptors at the nuclear membrane or perinuclear level are a new area of research. Some studies provide evidence for $EP_1$, $EP_4$, and/or $EP_{3\text{-}I}$ receptors at the perinuclear region in endothelial cells of porcine cerebral microvessel and in human embryonic kidney cells (HEK293)[3,57]. These results have shown that the activation of the nuclear $EP_{3\text{-}I}$ receptor could modulate iNOS and eNOS gene transcription[57,58]. In addition, they are consistent with the nuclear localization of COX[59], providing a local production of $PGE_2$, the preferential endogenous agonist for the EP receptors. The IP receptor may also be present at the nuclear level since PGI-synthase, the enzyme responsible for the conversion of $PGH_2$ into $PGI_2$, is also detected and colocalized at the perinuclear level with COX-1 in human endothelial cell line, ECV304[59].

PPARα, PPARβ (previously δ), and PPARγ are the second kind of nuclear receptor activated by prostanoids with numerous roles in vascular biology. The PPAR are activated by some natural derivatives of $PGD_2$ ($\Delta$12-$PGJ_2$, 15d-$PGJ_2$), in addition, PPARα and PPARβ may use prostacyclin as endogenous ligands[60]. However, there are many other endogenous PPAR activators, most of them are of lipidic origin and the predominant ligand remains to be determined for each PPAR subtype. Experiments of RT-PCR and western-blot showed that the PPAR are present in the HVSMC and endothelial cells in culture[60,61,62]. They are responsible for many physiological events, for example, the activation of PPARα receptors expressed in human aortic smooth muscle cells promotes their proliferation[59]. PPARγ has been systematically explored particularly with the synthetic agonists of the thiazolidinediones (TZD) class[60]. The activation of PPARγ *in vitro* inhibits the expression of various genes implicated in the proliferation, migration, and inflammatory response of the vascular cells[63]. On the other hand, in histological sections of human blood vessels, the PPARγ receptor is present in the endothelial cells, but absent in the smooth muscle[64,65].

The implication of PPARγ in vascular disease has been largely studied in atherosclerosis. The activation of this nuclear receptor controls the transcription of many genes (Ang II and $TxA_2$ receptors, Tx-synthase, ET-1, proteases, COX-2, and cytokines) having a role in vascular inflammation specifically at the level of the atherosclerotic lesions[60,66]. Immunohistochemistry studies using human coronary arteries obtained from recipients of heart transplants have shown that PPARγ is strongly expressed by the macrophages present in early and intermediate atherosclerotic lesions[66]. In these cells under inflammatory conditions, $PGJ_2$, Δ12-$PGJ_2$, 15d-$PGJ_2$, and TZD inhibit the expression of genes coding for proinflammatory cytokines (TNF-α, IL-1β, IL-6)[67] and for inducible enzymes (iNOS and COX-2)[60,68]. In the same way, the transcription of the matrix metalloproteinase-9 (MMP-9) gene as well as the migration of the HVSMC is inhibited after activation of the PPARγ receptor[61].

The activation of PPARγ plays an essentially antiatherosclerotic role, thus *in vivo,* the activation of PPARγ by the TZD significantly reduces the evolution of the atherosclerosis in humans[69,70,71]. Rosiglitazone protects the vascular wall by reducing proinflammatory responses and the occurrence of coronary events in type-2 diabetic patients within 6 months after percutaneous coronary intervention. Similarly, PPARα ligands like the fibrates decrease the risk of coronary heart diseases and the progression of premature atherosclerosis in patients with type-2 diabetes[60]. In addition, some dual PPARα/γ ligands have recently been developed and show a combined efficacy in the treatment of global risk in patients with the metabolic syndrome or type-2 diabetes[60].

CRTH2 is another prostanoid receptor activated by $PGD_2$ and its metabolites. This receptor has been recently described in human Th2 lymphocytes, Tc2 lymphocytes, eosinophil, and basophil[72]. On the other hand, neutrophils, platelets, Th1 lymphocytes, and B and T cells do not express the CRTH2 receptor on their surface[72,73,74]. CRTH2 receptor is activated selectively by DK-$PGD_2$[74], a natural metabolite of $PGD_2$[75], and also by the 11-dehydro-$TXB_2$, one of the principal metabolites of $TXA_2$[76]. In addition, 15d-$PGJ_2$, can bind to CRTH2 receptor with an equivalent affinity in comparison with $PGD_2$ and DK-$PGD_2$[74]. The eosinophil migration induced by the activation of CRTH2 receptor is blocked by the TP/CRTH2 antagonist BAY u3405 (ramatroban)[77]. Lastly, the activation of this receptor by $PGD_2$ decreases the intracellular production of cAMP, while an opposite effect is observed after activation of DP receptor, the preferential classical receptor for $PGD_2$[78,79].

The first studies carried out on humans concerning CRTH2 primarily implicated this receptor with the inflammatory allergic response[80]. One of the principal biological functions induced by the activation of this receptor is chemotaxis. Thus $PGD_2$ or DK-$PGD_2$ causes the migration of eosinophil, basophil, and the Th2 lymphocytes[72]. These events concern the pulmonary vessels during allergic or asthmatic responses, however, the role of CRTH2 in cardiovascular diseases has not been studied.

## PROSTANOID RECEPTORS IN HUMAN BLOOD CELLS

Blood cells and constitutive cells of the vascular wall permanently interact during healthy and pathological conditions. Two of these classical cell-cell interactions are the endothelial synthesis of $PGI_2$ from $PGH_2$ released by platelets and the synthesis of $TxA_2$ by platelets from $PGH_2$ released by the endothelial cells. These events are associated with hemostasis including the activation of prostanoid receptors present both in platelets and in cells of the vascular wall. Similar communication between blood cells and the endothelium exists during the inflammatory processes, implicating blood cell extravasations in vascular diseases, such as atherosclerosis, aneurysm, or during allergy and edema. Leukocyte rolling, adhesion, and migration through the endothelial cells are dependent on prostanoid receptor activation. For example, $PGE_2$ or $PGE_1$ can block the leukocyte migration through human endothelial cells in culture[81,82]. The different prostanoid receptors described in human blood cells and involved in these different events are indicated in Table 2.

**TABLE 2**
**Prostanoid Receptors in Human Blood Cells***

| Cells | Receptors | | Induced Effects (+ Activation, – Inhibition) | Ref. |
|---|---|---|---|---|
| Platelet | DP, IP | – | Aggregation | [83] |
| | TP, $EP_3$ | + | Aggregation | [83] |
| Erythrocyte | TP | + | Membrane destabilization | [84] |
| | IP | + | Insulin binding, membrane stabilization | [84,85] |
| Eosinophil | $EP_4$ | – | Production of $LTC_4$ | [86] |
| | DP | – | Apoptosis; adhesion | [73,78] |
| Neutrophil | $EP_2$, DP | – | $LTB_4$ and superoxide anion release | [87] |
| | DP | – | Migration | [88] |
| | FP | + | Migration | [30] |
| Basophil | IP, DP | + | cAMP production | [89] |
| Mastocyte | $EP_2$ | + | Vascular endothelial growth factor (VEGF) production | [90] |
| Monocyte | EP($_2$ or $_4$), IP | – | TNF-$\alpha$ production | [91] |
| | $EP_2$, $EP_4$ | + | Maturation in monocyte derived dentritic cell | [92] |
| | $EP_2$, $EP_4$ | – | Intercellular adhesion molecule-1 (ICAM-1; B7) expression | [93] |
| | EP($_2$ or $_4$) | + | COX-2 expression | [94] |
| | $EP_4$ | – | IL-12 production | [95] |
| | $EP_3$ | + | Migration | [96] |
| | IP | + | VEGF production | [97] |
| | TP | – | Migration, cell adhesion | [98] |
| Macrophage | $EP_4$ | – | Chemokine production | [99] |
| | $EP_4$ | + | IL-6 production | [4] |
| | EP($_2$ or $_4$) | + | Migration | [100] |
| B Lymphocyte | $EP_4$ | | $EP_4$ mRNA expression | [101] |
| T Lymphocyte | $EP_3$ | + | Matrix metalloproteinase 9 (MMP-9) production | [102] |
| | $EP_4$ | – | IL-2 production | [103] |
| | $EP_4$ | + | IL-6 production | [104] |
| | $EP_2$ | – | Apoptosis | [105] |

* Prostanoid receptors described in human blood cells and the induced effects after their stimulation. (Receptors absent or not determined were not distinguished.)

## ANGIOGENESIS AND THE PROSTANOID RECEPTORS

The proliferation of vascular smooth muscle cells (HVSMC) derived from the human pulmonary artery induced by fetal calf serum is inhibited by $PGI_2$, $PGE_2$, or cicaprost [41,48,106]. These results suggest activation of the IP receptor and probably that of an EP receptor during this effect. The activation of IP receptor by iloprost also reduces the migration induced by endothelial cell–conditioned medium as chemoattractant of the HVSMC derived from mammary artery[47]. A recent study based on microarray[107] confirmed the inhibitory role of IP receptors in the proliferation and the migration of the HVSMC. These authors showed that stimulation by iloprost modifies the expression of 83 genes in the HVSMC. Some of these genes (zinc finger transcription factor [hEZF], growth arrest specific gene 1

[gas1], VEGF, cysteine-rich angiogenic protein [Cyr61]) are known to be implicated in smooth muscle growth and cell migration[107,108].

The incubation of aorta HVSMC with $PGE_2$ decreased the cellular proliferation and the DNA synthesis[109]. These authors suggested the involvement of the $EP_1$ receptors since this inhibition was observed with $PGE_2$ while $PGE_1$ was ineffective. The pharmacological characterization of this receptor remains to be confirmed since another study showed that $PGE_1$ and $PGE_2$ inhibited the growth of these cells[110]. In addition, the agonists of the EP receptors can also control the migration of HVSMC. The migration of HVSMC derived from pulmonary artery induced by platelet-derived growth factor (PDGF) is inhibited by $PGE_2$[111]. $PGE_2$ elicited this effect with an efficacy associated with its ability to promote cAMP accumulation and PKA activation. This intracellular signaling suggests an inhibition of the migration mediated by an $EP_{2/4}$ receptor. On the contrary, M&B28767, the selective agonist for $EP_3$ receptors, stimulates the migration of the HVSMC derived from the mammary artery[47]. Finally, similar control of the endothelial cell chemotaxis by $PGE_2$ and thromboxane has been described; the HUVEC migration induced by IL-1β are partially blocked by $EP_4$, or TP-selective antagonists[112].

The COX-2 activity participates in angiogenesis observed in different models, such as rodent corneas[112,113], proliferation of human endothelial cell culture[114], or in pathophysiological conditions, such as diabetic and ischemic retinopathy[115]. Prostanoids derived from the COX-2 activity play a key role in oncogenesis since an increased expression of this enzyme has been detected in colon, breast, gastric, lung, and pancreatic cancers[116]. More specifically, the association of the COX-2 activity with angiogenesis in different forms of human cancer is well documented. An elevated COX-2 expression is detected with lymph node metastases and reduced survival in Barrett's cancer. This event appears to be related to the induction of angiogenesis and proliferation[117]. The COX-2 expression correlates with microvessel density and VEGF production in the angiogenesis of human non-small cell lung cancer[118]. Hepatocellular carcinoma is a highly malignant tumor characterized by active neovascularization; the VEGF production and venous invasion correlate with COX-2 activity[119]. The COX-2 inhibitors reduce tumor induced angiogenesis when xenografts of human colon cancer cells are planted in murine hosts[113]. However, the prostanoid receptors involved in human tumor angiogenesis are not known, while the $EP_3$ subtype has been suggested in mice models of angiogenesis and tumor growth[120].

## PROSTANOID RECEPTORS INVOLVED IN THE HUMAN VASCULAR TONE

When the receptors are localized on the smooth muscle, the activation of IP, $EP_2$, $EP_4$, or DP receptors by prostanoids induces vasodilatation, while the activation of TP, $EP_1$, $EP_3$, or FP receptors is responsible for vasoconstrictions[121]. Most of the studies on vascular tone (Table 3) describe contractions produced by the $TxA_2$ analog (U46619) and relaxations induced by the $PGI_2$ analogs (iloprost or cicaprost). These results indicate that most human vascular smooth muscle expresses TP and IP receptors (Table 3). However, in these studies, the involvement of the IP receptor should be considered with caution since iloprost and, particularly, cicaprost have recently been described as potent agonists for the $EP_4$ receptor subtype[122,123,124].

There are very few studies concerning the involvement of other prostanoid receptor subtypes (EP, FP, or DP; Table 3) in the control of the vascular tone, although there is increasing evidence for the presence and a role for the four EP receptor subtypes preferentially stimulated by $PGE_2$[125,126,127,128,129,130]. In addition, a characterization of the EP receptors should be of interest since there is a striking increase in the quantities of the inducible mPGES-1 under inflammatory conditions and, in consequence, an increased concentration of $PGE_2$ in the vascular wall or in the blood of atherosclerosis patients[131,132].

**TABLE 3**
**Prostanoid Receptors Involved in the Control (*In Vivo* or *In Vitro*) of Human Vascular Smooth Muscle Tone***

| Vascular Preparations | Receptor Involved in | | Ref. |
|---|---|---|---|
| | Vasoconstriction | Vasodilatation | |
| Basilar artery | TP, $EP_?$ | IP | [133] |
| Cerebral artery | TP | IP, $EP_4$ | [127] |
| Pial artery | TP | IP | [134,135] |
| Retinal arteriole | TP | DP | [136] |
| Coronary artery | TP | IP | [137,138,139] |
| Mammary artery | TP | | [51] |
| Thymic artery | TP | | [140] |
| Pulmonary artery | TP, $EP_3$ | IP | [128,130,141,142,143] |
| Pulmonary vein | TP, $EP_1$ | IP, DP, $EP_?$ | [129,130] |
| Gastroepiploic artery | TP | | [49,51] |
| Gastroepiploic vein | TP | | [49] |
| Mesenteric artery | | IP | [138] |
| Brachial artery | | IP | [144] |
| Radial artery | TP | | [51] |
| Hand artery | | IP | [145] |
| Hand vein | TP | IP | [145,146] |
| Uterine artery | TP | IP, $EP_4$ | [126] |
| Placental artery | TP | | [147] |
| Placental vein | TP | | [148] |
| Umbilical artery | TP | IP | [147,149] |
| Umbilical vein | TP, FP | | [147,148,150] |
| Penile artery | TP | IP, $EP_?$ | [125] |
| Femoral artery | TP | | [151] |
| Saphenous vein | TP | | [152] |

* In this table, only the prostanoid receptor subtypes described in the literature are indicated. $EP_?$ Indicates that a specific EP response to $PGE_2$ was observed, however, the EP subtype has not been characterized.

## VASCULAR PATHOLOGIES AND THE CLASSICAL PROSTANOID RECEPTORS

The IP agonists — the synthetic $PGI_2$ (epoprostenol), $PGE_1$ (alprostadil), or the $PGI_2$ analogue iloprost — have been used for the last 20 years in the treatment of human pulmonary hypertension. These compounds decrease the pulmonary vascular resistance and may be administered by inhalation (iloprost) or continuous subcutaneous infusion, as in the case of treprostinil, the last $PGI_2$ analogue synthesized[153]. Intravenous injection of $PGE_1$ is also useful to maintain the patency of the ductus arteriosus in infants with certain cardiac malformations[5]. Similar to the treatment of these pathologies, in the near future, the prostanoid receptors will probably be new therapeutic targets for the treatment of other cardiovascular diseases. Changes in the density or appearance of new prostanoid receptor subtypes and isoforms in the vascular wall are associated with the development of cardiovascular pathologies.

This variation of receptorial expression is observed during atherosclerosis, for example, where the density of TP receptor is increased in the atherosclerotic coronary artery[46]. The $EP_4$ receptor is detected by immunohistochemistry in human carotid atherosclerotic plaques, in macrophage-rich lesions, and in smooth muscle, while no staining was observed in the vascular wall of normal carotid artery[99]. The $EP_2$ receptor has also been detected in these *atheroma* plaques[154]. In addition, in this study on HVSMC derived from the media of human internal mammary arteries, the activation of $EP_2$ and IP receptors induced an up-regulation of hyaluronic acid. This compound is a prominent constituent of the extracellular matrix in atherosclerotic vascular lesions in humans and is known to modulate the vascular smooth muscle phenotype. However, the transcription and expression of the $EP_4$ receptor in the human carotid atherosclerotic plaques are largely predominant in comparison to the $EP_2$ subtype[155]. In addition, a stronger expression and a greater detection was observed for the $EP_4$ receptor in the carotid atherosclerotic plaque of symptomatic patients vs. the asymptomatic ones[155]. The $EP_4$ receptor localized mainly in the macrophage-rich lesions, together with COX-2, mPGES-1, MMP-9, and MMP-2 [45,99,155]. The activation of this receptor by $PGE_2$ and the subsequent production of MMP probably contribute to the plaque destabilization.

In the previous studies, the appearance of the prostanoid receptors in the atherosclerotic vascular wall may be due to the migration of macrophages expressing these receptors and/or an induction of their expression by the constitutive cells of the vascular wall. As in the case of the inducible enzymes (COX-2, mPGES-1), it is not excluded that the proinflammatory conditions found in atherosclerosis may also stimulate the transcription of prostanoid receptors in the vascular cells. This induction has been observed with the increased expression of $EP_2$ and $EP_4$ receptors in human nonvascular cells, as occurs in the experiments with the synovial and cervical fibroblasts treated with IL-1β[156,157].

$PGE_2$ receptors in abdominal aortic aneurysm have been explored[4]. This study shows that the release of IL-6 by the aortic aneurysm wall is more specifically due to the activation of the $EP_4$ receptor present in the macrophages instead of the smooth muscle cells. The production of this proinflammatory cytokine mediates aneurysmal degeneration. The level of IL-6 measured postsurgery in the blood of patients with ruptured abdominal aortic aneurysms was significantly increased in nonsurvivors vs. survivors[158].

Another $PGE_2$ receptor, namely, the $EP_3$ receptor subtype, is implicated in the development of vascular pathologies. The DeCODE Company has developed an $EP_3$ antagonist (NG041) presently in phase IIa clinical trial. This compound is an example of the new drugs targeting the prostanoid receptors. Through their population genetic research in Iceland, this company has identified common versions of the gene encoding the $EP_3$ receptor that confer increasing risk of atherosclerosis[159]. These epidemiological data are in accordance with the different proatherogenic roles of the $EP_3$ receptor in human tissues, that is, vasoconstriction[128,160], migration of vascular smooth muscle cells[47], and platelet activation[83].

In a general way, the prostanoid receptor ligands used in cardiovascular therapeutics should at least stimulate the vasodilatation, block the vasoconstriction and inhibit the platelet aggregation. More specifically they should activate the IP- and DP- receptors and block the TP- and $EP_3$- receptors since these receptors are also present on human platelet[83]. In addition, from the data previously described in atherosclerotic and aneurismal tissues, development of specific antagonists for the receptors involved in the inflammatory processes of the vascular wall, such as $EP_4$, should be also useful.

## CONCLUSION

After the cardiovascular risks encountered with the COX-2 inhibitors, new and more selective strategies have been developed to modulate the physiological effects mediated by prostanoids. The different prostanoid receptors, as well as the different enzymes of the cyclooxygenase pathway, such as the prostanoid synthases, will be future therapeutic targets for the treatment of cardiovascular pathologies. The PPARs have already become therapeutic targets through the use of the fibrate class and the insulin-sensitizing thiazolidinediones. These compounds are used to reduce the cardiovascular risk in patients with atherosclerosis, metabolic syndrome, and/or diabetes. Furthermore, the different physiological roles of the classical prostanoid receptor subtypes and isoforms, the nuclear receptors, and the CRTH2 in the human vascular wall are intensively explored. In addition to $PGI_2$ and thromboxane, there is increasing evidence that suggests a key cardiovascular role for $PGE_2$. The four EP receptor subtypes activated by $PGE_2$ are present on the cells of the vascular wall, as well as in the blood cell during vascular inflammation. Their activation and, more specifically, the $EP_3$ and $EP_4$ receptor activation appears associated in most human physiological or pathophysiological responses of the vascular wall. For these reasons, these classical prostanoid receptors are also promising cardiovascular therapeutic targets.

## References

1. Kotani, M., Tanaka, I., Ogawa, Y., Usui, T., Tamura, N., Mori, K., Narumiya, S., Yoshimi, T., and Nakao, K. (1997) Structural organization of the human prostaglandin EP3 receptor subtype gene (PTGER3). *Genomics* **40(3),** 425–434.
2. Fujino, H. and Regan, J.W. (2003) Prostanoid receptors and phosphatidylinositol 3-kinase: a pathway to cancer? *Trends Pharmacol. Sci.* **24(7)**, 335–340.
3. Zhu, T., Gobeil, F., Vazquez-Tello, A., Leduc, M., Rihakova, L., Bossolasco, M., Bkaily, G., Peri, K., Varma, D.R., Orvoine, R., and Chemtob, S. (2006) Intracrine signaling through lipid mediators and their cognate nuclear G-protein-coupled receptors: a paradigm based on PGE2, PAF, and LPA1 receptors. *Can. J. Physiol. Pharmacol.* **84(3–4)**, 377–391.
4. Bayston, T., Ramessur, S., Reise, J., Jones, K.G., and Powell, J.T. (2003) Prostaglandin E2 receptors in abdominal aortic aneurysm and human aortic smooth muscle cells. *J. Vasc. Surg.* **38(2),** 354–359.
5. Leonhardt, A., Glaser, A., Wegmann, M., Schranz, D., Seyberth, H., and Nusing, R. (2003) Expression of prostanoid receptors in human ductus arteriosus. *Br. J. Pharmacol.* **138(4),** 655–659.
6. Paul, B.Z., Ashby, B., and Sheth, S.B. (1998) Distribution of prostaglandin IP and EP receptor subtypes and isoforms in platelets and human umbilical artery smooth muscle cells. *Br. J. Haematol.* **102(5)**, 1204–1211.
7. Boie, Y., Sawyer, N., Slipetz, D.M., Metters, K.M., and Abramovitz, M. (1995) Molecular cloning and characterization of the human prostanoid DP receptor. *J. Biol. Chem.* **270(32)**, 18910–18916.
8. Funk, C.D., Furci, L., FitzGerald, G.A., Grygorczyk, R., Rochette, C., Bayne, M.A., Abramovitz, M., Adam, M., and Metters, K.M. (1993) Cloning and expression of a cDNA for the human prostaglandin E receptor EP1 subtype. *J. Biol. Chem.* **268(35)**, 26767–26772.
9. Regan, J.W., Bailey, T.J., Pepperl, D.J., Pierce, K.L., Bogardus, A.M., Donello, J.E., Fairbairn, C.E., Kedzie, K.M., Woodward, D.F., and Gil, D.W. (1994) Cloning of a novel human prostaglandin receptor with characteristics of the pharmacologically defined EP2 subtype. *Mol. Pharmacol.* **46(2)**, 213–220.
10. An, S., Yang, J., Xia, M., and Goetzl, E.J. (1993) Cloning and expression of the EP2 subtype of human receptors for prostaglandin E2. *Biochem. Biophys. Res. Commun.* **197(1),** 263–270.
11. Abramovitz, M., Boie, Y., Nguyen, T., Rushmore, T.H., Bayne, M.A., Metters, K.M., Slipetz, D.M., and Grygorczyk, R. (1994) Cloning and expression of a cDNA for the human prostanoid FP receptor. *J. Biol. Chem.* **269(4)**, 2632–2636.
12. Boie, Y., Rushmore, T.H., Darmon-Goodwin, A., Grygorczyk, R., Slipetz, D.M., Metters, K.M., and Abramovitz, M. (1994) Cloning and expression of a cDNA for the human prostanoid IP receptor. *J. Biol. Chem.* **269(16),** 12173–12178.
13. Hirata, M., Hayashi, Y., Ushikubi, F., Yokota, Y., Kageyama, R., Nakanishi, S., and Narumiya, S. (1991) Cloning and expression of cDNA for a human thromboxane A2 receptor. *Nature* **349(6310),** 617–620.
14. Narumiya, S., Sugimoto, Y., and Ushikubi, F. (1999) Prostanoid receptors: structures, properties, and functions. *Physiol. Rev.* **79(4),** 1193–1226.
15. Tsuboi, K., Sugimoto, Y., and Ichikawa, A. (2002) Prostanoid receptor subtypes. *Prostaglandins Other Lipid Mediat.* **68–69,** 535–556.
16. Alhenc-Gelas, F., Tsai, S.J., Callahan, K.S., Campbell, W.B., and Johnson, A.R. (1982) Stimulation of prostaglandin formation by vasoactive mediators in cultured human endothelial cells. *Prostaglandins* **24(5),** 723–742.
17. McIntyre, T.M., Zimmerman, G.A., Satoh, K., and Prescott, S.M. (1985) Cultured endothelial cells synthesize both platelet-activating factor and prostacyclin in response to histamine, bradykinin, and adenosine triphosphate. *J. Clin. Invest.* **76(1),** 271–280.
18. Walch, L., Gascard, J.P., Dulmet, E., Brink, C., and Norel, X. (2000) Evidence for a M(1) muscarinic receptor on the endothelium of human pulmonary veins. *Br. J. Pharmacol.* **130(1),** 73–78.
19. Okatani, Y., Watanabe, K., Nakano, Y., and Sagara, Y. (1996) Relaxant effect of nitric oxide and prostacyclin on serotonin-induced vasocontraction of human umbilical artery. *Acta Obstet. Gynecol. Scand.* **75(2),** 108–112.
20. Norel, X., Walch, L., Costantino, M., Labat, C., Gorenne, I., Dulmet, E., Rossi, F., and Brink, C. (1996) M1 and M3 muscarinic receptors in human pulmonary arteries. *Br. J. Pharmacol.* **119(1),** 149–157.
21. Norel, X. and Brink, C. (2004) The quest for new cysteinyl-leukotriene and lipoxin receptors: recent clues. *Pharmacol. Ther.* **103(1),** 81–94.
22. Brandt, R., Dembinska-Kiec, A., Korbut, R., Gryglewski, R.J., and Nowak, J. (1984) Release of prostacyclin from the human pulmonary vascular bed in response to cholinergic stimulation. *Naunyn Schmiedebergs Arch. Pharmacol.* **325(1)**, 69–75.
23. Norel, X., Haye-Legrand, I., Labat, C., Benveniste, J., and Brink, C. (1991) Antigen-induced contraction of human isolated lung preparations passively sensitized with monoclonal IgE: effects of indomethacin. *Int. Arch. Allergy Appl. Immunol.* **96(4),** 368–375.
24. Dore, M., Cote, L.C., Mitchell, A., and Sirois, J. (1998) Expression of prostaglandin G/H synthase type 1, but not type 2, in human ovarian adenocarcinomas. *J. Histochem. Cytochem.* **46(1),** 77–84.
25. Yang, X., Sheares, K.K., Davie, N., Upton, P.D., Taylor, G.W., Horsley, J., Wharton, J., and Morrell, N.W. (2002) Hypoxic induction of cox-2 regulates proliferation of human pulmonary artery smooth muscle cells. *Am. J. Respir. Cell Mol. Biol.* **27(6),** 688–696.
26. Norel, X., Walch, L., Gascard, J.P., deMontpreville, V., and Brink, C. (2004) Prostacyclin release and receptor activation: differential control of human pulmonary venous and arterial tone. *Br. J. Pharmacol.* **142(4),** 788–796.
27. Schonbeck, U., Sukhova, G.K., Graber, P., Coulter, S., and Libby, P. (1999) Augmented expression of cyclooxygenase-2 in human atherosclerotic lesions. *Am. J. Pathol.* **155(4),** 1281–1291.
28. Spisni, E., Bianco, M.C., Griffoni, C., Toni, M., D'Angelo, R., Santi, S., Riccio, M., and Tomasi. V. (2003) Mechanosensing role of caveolae and caveolar constituents in human endothelial cells. *J. Cell Physiol.* **197(2),** 198–204.
29. Topper, J.N., Cai, J., Falb, D., and Gimbrone, M.A., Jr. (1996) Identification of vascular endothelial genes differentially responsive to fluid mechanical stimuli: cyclooxygenase-2, manganese superoxide dismutase, and endothelial cell nitric oxide synthase are selectively up-regulated by steady laminar shear stress. *Proc. Natl. Acad. Sci. U. S. A.* **93(19)**, 10417–10422.
30. Arnould, T., Thibaut-Vercruyssen, R., Bouaziz, N., Dieu, M., Remacle, J., and Michiels, C. (2001) PGF(2alpha), a prostanoid released by endothelial cells activated by hypoxia, is a chemoattractant candidate for neutrophil recruitment. *Am. J. Pathol.* **159(1),** 345–357.
31. Ristimaki, A. and Viinikka, L. (1992) Modulation of prostacyclin production by cytokines in vascular endothelial cells. *Prostaglandins Leukot. Essent. Fatty Acids* **47(2),** 93–99.
32. Mitchell, M.D., Romero, R.J., Lepera, R., Rittenhouse, L., and Edwin, S.S. (1990) Actions of endothelin-1 on prostaglandin production by gestational tissues. *Prostaglandins* **40(6),** 627–635.
33. Gimbrone, M.A., Jr. and Alexander, R.W. (1975) Angiotensin II stimulation of prostaglandin production in cultured human vascular endothelium. *Science* **189(4198),** 219–220.
34. Cabre, F., Tost, D., Suesa, N., Gutierrez, M., Ucedo, P., Mauleon, D., and Carganico, G. (1993) Synthesis and release of platelet-activating factor and eicosanoids in human endothelial cells induced by different agonists. *Agents Actions* **38(3–4)**, 212–219.
35. Maier, J.A., Hla, T., and Maciag, T. (1990) Cyclooxygenase is an immediate-early gene induced by interleukin-1 in human endothelial cells. *J. Biol. Chem.* **265(19)**, 10805–10808.
36. Jones, D.A., Carlton, D.P., McIntyre, T.M., Zimmerman, G.A., and Prescott, S.M. (1993) Molecular cloning of human prostaglandin endoperoxide synthase type II and demonstration of expression in response to cytokines. *J. Biol. Chem.* **268(12)**, 9049–9054.
37. Uracz, W., Uracz, D., Olszanecki, R., and Gryglewski, R.J. (2002) Interleukin 1beta induces functional prostaglandin E synthase in cultured human umbilical vein endothelial cells. *J. Physiol. Pharmacol.* **53(4 Pt 1),** 643–654.
38. Soler, M., Camacho, M., Escudero, J.R., Iniguez, M.A., and Vila, L. (2000) Human vascular smooth muscle cells but not endothelial cells express prostaglandin E synthase. *Circ. Res.* **87(6),** 504–507.
39. Wen, F.Q., Watanabe, K., and Yoshida, M. (1998) Eicosanoid profile in cultured human pulmonary artery smooth muscle cells treated with IL-1 beta and TNF alpha. *Prostaglandins Leukot. Essent. Fatty Acids* **59(1),** 71–75.
40. Yamamoto, M., Aoyagi, M., Fukai, N., Matsushima, Y., and Yamamoto, K. (1999) Increase in prostaglandin E(2) production by interleukin-1beta in arterial smooth muscle cells derived from patients with moyamoya disease. *Circ. Res.* **85(10)**, 912–918.
41. Jourdan, K.B., Evans, T.W., Lamb, N.J., Goldstraw, P., and Mitchell, J.A. (1999) Autocrine function of inducible nitric oxide synthase and cyclooxygenase-2 in proliferation of human and rat pulmonary artery smooth-muscle cells: species variation. *Am. J. Respir. Cell Mol. Biol.* **21(1)**, 105–110.
42. Wen, F.Q., Watanabe, K., Tanaka, H., and Yoshida, M. (1997) Cytokines and lipopolysaccharide enhance basal and thrombin-stimulated production of PGI2 by cultured human pulmonary artery smooth muscle cells. *Prostaglandins Leukot. Essent. Fatty Acids* **56(3),** 185–192.
43. Wen, F.Q., Watanabe, K., and Yoshida, M. (1997) Lipopolysaccharide and cytokines enhance bradykinin-stimulated production of PGI2 by cultured human pulmonary artery smooth muscle cells. *Cell Biol. Int.* **21(5),** 321–327.
44. Zhang, Y., Cao, H.J., Graf, B., Meekins, H., Smith, T.J., and Phipps, R.P. (1998) CD40 engagement up-regulates cyclooxygenase-2 expression and prostaglandin E2 production in human lung fibroblasts. *J. Immunol.* **160(3),** 1053–1057.
45. Cipollone, F., Rocca, B., and Patrono, C. (2004) Cyclooxygenase-2 expression and inhibition in atherothrombosis. *Arterioscler. Thromb. Vasc. Biol.* **24**, 1–10.
46. Katugampola, S.D. and Davenport, A.P. (2001) Thromboxane receptor density is increased in human cardiovascular disease with evidence for inhibition at therapeutic concentrations by the AT(1) receptor antagonist losartan. *Br. J. Pharmacol.* **134(7)**, 1385–1392.

47. Blindt, R., Bosserhoff, A.K., vom Dahl, J., Hanrath, P., Schror, K., Hohlfeld, T., and Meyer-Kirchrath, J. (2002) Activation of IP and EP(3) receptors alters cAMP-dependent cell migration. *Eur. J. Pharmacol.* **444(1–2),** 31–37.
48. Wharton, J., Davie, N., Upton, P.D., Yacoub, M.H., Polak, J.M., and Morrell, N.W. (2000) Prostacyclin analogues differentially inhibit growth of distal and proximal human pulmonary artery smooth muscle cells. *Circulation* **102(25),** 3130–3136.
49. Wallerstedt, S.M. and Bodelsson, M. (1997) Endothelium-dependent relaxation by substance P in human isolated omental arteries and veins: relative contribution of prostanoids, nitric oxide and hyperpolarization. *Br. J. Pharmacol.* **120(1),** 25–30.
50. Malomvolgyi, B., Hadhazy, P., and Magyar, K. (1988) Relaxation by prostacyclin (PGI2) of human, dog and rabbit femoral artery strips. Interspecies difference. *Biomed. Biochim. Acta* **47(10–11),** S125–128.
51. Chardigny, C., Jebara, V.A., Acar, C., Descombes, J.J., Verbeuren, T.J., Carpentier, A., and Fabiani, J.N. (1993) Vasoreactivity of the radial artery. Comparison with the internal mammary and gastroepiploic arteries with implications for coronary artery surgery. *Circulation* **88(5 Pt 2),** II115–127.
52. Norel, X., Walch, L., and Brink, C. (2002) U46619 induced contractions: role of prostanoids and nitric oxide in human pulmonary vessels. *Br. J. Pharmacol.* Proc Sup, P064.
53. Ashton, A.W. and Ware, J.A. (2004) Thromboxane A2 receptor signaling inhibits vascular endothelial growth factor-induced endothelial cell differentiation and migration. *Circ. Res.* **95(4),** 372–379.
54. Nie, D., Lamberti, M., Zacharek, A., Li, L., Szekeres, K., Tang, K., Chen, Y., and Honn, K.V. (2000) Thromboxane A(2) regulation of endothelial cell migration, angiogenesis, and tumor metastasis. *Biochem. Biophys. Res. Commun.* **267(1),** 245–251.
55. Daniel, T.O., Liu, H., Morrow, J.D., Crews, B.C., and Marnett, L.J. (1999) Thromboxane A2 is a mediator of cyclooxygenase-2-dependent endothelial migration and angiogenesis. *Cancer Res.* **59(18),** 4574–4577.
56. Arner, M., Uski, T., and Hogestatt, E.D. (1994) Endothelium dependence of prostanoid-induced relaxation in human hand veins. *Acta Physiol. Scand.* **150(3),** 267–272.
57. Bhattacharya, M., Peri, K., Ribeiro-da-Silva, A., Almazan, G., Shichi, H., Hou, X., Varma, D.R., and Chemtob, S. (1999) Localization of functional prostaglandin E2 receptors EP3 and EP4 in the nuclear envelope. *J. Biol. Chem.* **274(22),** 15719–15724.
58. Gobeil, F., Jr., Dumont, I., Marrache, A.M., Vazquez-Tello, A., Bernier, S.G., Abran, D., Hou, X., Beauchamp, M.H., Quiniou, C., Bouayad, A., Choufani, S., Bhattacharya, M., Molotchnikoff, S., Ribeiro-Da-Silva, A., Varma, D.R., Bkaily, G., and Chemtob, S. (2002) Regulation of eNOS expression in brain endothelial cells by perinuclear EP(3) receptors. *Circ. Res.* **90(6),** 682–689.
59. Lim, H. and Dey, S.K. (2002) A novel pathway of prostacyclin signaling-hanging out with nuclear receptors. *Endocrinology* **143(9),** 3207–3210.
60. Moraes, L.A., Piqueras, L., and Bishop-Bailey, D. (2006) Peroxisome proliferator-activated receptors and inflammation. *Pharmacol. Ther.* **110(3),** 371–385.
61. Marx, N., Schonbeck, U., Lazar, M.A., Libby, P., and Plutzky, J. (1998) Peroxisome proliferator-activated receptor gamma activators inhibit gene expression and migration in human vascular smooth muscle cells. *Circ. Res.* **83(11),** 1097–1103.
62. Goetze, S., Eilers, F., Bungenstock, A., Kintscher, U., Stawowy, P., Blaschke, F., Graf, K., Law, R.E., Fleck, E., and Grafe, M. (2002) PPAR activators inhibit endothelial cell migration by targeting Akt. *Biochem. Biophys. Res. Commun.* **293(5),** 1431–1437.
63. Bishop-Bailey, D. (2000) Peroxisome proliferator-activated receptors in the cardiovascular system. *Br. J. Pharmacol.* **129(5),** 823–834.
64. Marx, N., Bourcier, T., Sukhova, G.K., Libby, P., and Plutzky, J. (1999) PPARgamma activation in human endothelial cells increases plasminogen activator inhibitor type-1 expression: PPARgamma as a potential mediator in vascular disease. *Arterioscler. Thromb. Vasc. Biol.* **19(3),** 546–551.
65. Plutzky, J. (1999) Atherosclerotic plaque rupture: emerging insights and opportunities. *Am. J. Cardiol.* **84(1A),** 15J–20J.
66. Ricote, M., Huang, J., Fajas, L., Li, A., Welch, J., Najib, J., Witztum, J.L., Auwerx, J., Palinski, W., and Glass, C.K. (1998) Expression of the peroxisome proliferator-activated receptor gamma (PPARgamma) in human atherosclerosis and regulation in macrophages by colony stimulating factors and oxidized low density lipoprotein. *Proc. Natl. Acad. Sci. U. S. A.* **95(13),** 7614–7619.
67. Jiang, C., Ting, A.T., and Seed, B. (1998) PPAR-gamma agonists inhibit production of monocyte inflammatory cytokines. *Nature* **391(6662),** 82–86.
68. Takata, Y., Kitami, Y., Yang, Z.H., Nakamura, M., Okura, T., and Hiwada, K. (2002) Vascular inflammation is negatively autoregulated by interaction between CCAAT/enhancer-binding protein-delta and peroxisome proliferator-activated receptor-gamma. *Circ. Res.* **91(5),** 427–433.
69. Haffner, S.M., Greenberg, A.S., Weston, W.M., Chen, H., Williams, K., and Freed, M.I. (2002) Effect of rosiglitazone treatment on nontraditional markers of cardiovascular disease in patients with type 2 diabetes mellitus. *Circulation* **106(6),** 679–684.
70. Koshiyama, H., Shimono, D., Kuwamura, N., Minamikawa, J., and Nakamura, Y. (2001) Rapid communication: inhibitory effect of pioglitazone on carotid arterial wall thickness in type 2 diabetes. *J. Clin. Endocrinol. Metab.*

**86(7),** 3452–3456.

71. Minamikawa, J., Tanaka, S., Yamauchi, M., Inoue, D., and Koshiyama, H. (1998) Potent inhibitory effect of troglitazone on carotid arterial wall thickness in type 2 diabetes. *J. Clin. Endocrinol. Metab.* **83(5),** 1818–1820.
72. Hirai, H., Tanaka, K., Takano, S., Ichimasa, M., Nakamura, M., and Nagata, K. (2002) Cutting edge: agonistic effect of indomethacin on a prostaglandin D2 receptor, CRTH2. *J. Immunol.* **168(3),** 981–985.
73. Monneret, G., Gravel, S., Diamond, M., Rokach, J., and Powell, W.S. (2001) Prostaglandin D2 is a potent chemoattractant for human eosinophils that acts via a novel DP receptor. *Blood* **98(6),** 1942–1948.
74. Sawyer, N., Cauchon, E., Chateauneuf, A., Cruz, R.P., Nicholson, D.W., Metters, K.M., O'Neill, G.P, and Gervais, F.G. (2002) Molecular pharmacology of the human prostaglandin D2 receptor, CRTH2. *Br. J. Pharmacol.* **137(8),** 1163–1172.
75. Monneret, G., Cossette, C., Gravel, S., Rokach, J., and Powell, W.S. (2003) 15R-methyl-prostaglandin D2 is a potent and selective CRTH2/DP2 receptor agonist in human eosinophils. *J. Pharmacol. Exp. Ther.* **304(1),** 349–355.
76. Bohm, E., Sturm, G.J., Weiglhofer, I., Sandig, H., Shichijo, M., McNamee, A., Pease, J.E., Kollroser, M., Peskar, B.A., and Heinemann, A. (2004) 11-Dehydro-thromboxane B2, a stable thromboxane metabolite, is a full agonist of chemoattractant receptor-homologous molecule expressed on TH2 cells (CRTH2) in human eosinophils and basophils. *J. Biol. Chem.* **279(9),** 7663–7670.
77. Sugimoto, H., Shichijo, M., Iino, T., Manabe, Y., Watanabe, A., Shimazaki, M., Gantner, F., and Bacon, K.B. (2003) An orally bioavailable small molecule antagonist of CRTH2, ramatroban (BAY u3405), inhibits prostaglandin D2-induced eosinophil migration in vitro. *J. Pharmacol. Exp. Ther.* **305(1),** 347–352.
78. Gervais, F.G., Cruz, R.P., Chateauneuf, A., Gale, S., Sawyer, N., Nantel, F., Metters, K.M., and O'Neill, G.P. (2001) Selective modulation of chemokinesis, degranulation, and apoptosis in eosinophils through the PGD2 receptors CRTH2 and DP. *J. Allergy Clin. Immunol.* **108(6),** 982–988.
79. Trist, D.G., Collins, B.A., Wood, J., Kelly, M.G., and Robertson, A.D. (1989) The antagonism by BW A868C of PGD2 and BW245C activation of human platelet adenylate cyclase. *Br. J. Pharmacol.* **96(2),** 301–306.
80. Romagnani, S. (2002) Cytokines and chemoattractants in allergic inflammation. *Mol. Immunol.* **38(12–13),** 881–885.
81. Lou, J., Buhler, L., Deng, S., Mentha, G., Montesano, R., Grau, G.E., and Morel, P. (1998) Inhibition of leukocyte adherence and transendothelial migration in cultured human liver vascular endothelial cells by prostaglandin E1. *Hepatology* **27(3),** 822–828.
82. Oppenheimer-Marks, N., Kavanaugh, A.F., and Lipsky, P.E. (1994) Inhibition of the transendothelial migration of human T lymphocytes by prostaglandin E2. *J. Immunol.* **152(12),** 5703–5713.
83. Armstrong, R.A. (1996) Platelet prostanoid receptors. *Pharmacol. Ther.* **72(3),** 171–191.
84. Brezinski, M.E., Lefer, D.J., Bowker, B., and Lefer, A.M. (1987) Thromboxane induced red blood cell lysis. *Prostaglandins* **33(1),** 75–84.
85. Dutta-Roy, A.K., Kahn, N.N., and Sinha, A.K. (1991) Interaction of receptors for prostaglandin E1/prostacyclin and insulin in human erythrocytes and platelets. *Life Sci.* **49(16),** 1129–1139.
86. Mita, H., Hasegawa, M., Higashi, N., and Akiyama, K. (2002) Characterization of PGE2 receptor subtypes in human eosinophils. *J. Allergy Clin. Immunol.* **110(3),** 457–459.
87. Wheeldon, A. and Vardey, C.J. (1993) Characterization of the inhibitory prostanoid receptors on human neutrophils. *Br. J. Pharmacol.* **108(4),** 1051–1054.
88. Armstrong, R.A. (1995) Investigation of the inhibitory effects of PGE2 and selective EP agonists on chemotaxis of human neutrophils. *Br. J. Pharmacol.* **116(7),** 2903–2908.
89. Virgolini, I., Li, S., Sillaber, C., Majdic, O., Sinzinger, H., Lechner, K., Bettelheim, P., and Valent, P. (1992) Characterization of prostaglandin (PG)-binding sites expressed on human basophils. Evidence for a prostaglandin E1, I2, and a D2 receptor. *J. Biol. Chem.* **267(18),** 12700–12708.
90. Abdel-Majid, R.M. and Marshall, J.S. (2004) Prostaglandin E2 induces degranulation-independent production of vascular endothelial growth factor by human mast cells. *J. Immunol.* **172(2),** 1227–1236.
91. Meja, K.K., Barnes, P.J., and Giembycz, M.A. (1997) Characterization of the prostanoid receptor(s) on human blood monocytes at which prostaglandin E2 inhibits lipopolysaccharide-induced tumour necrosis factor-alpha generation. *Br. J. Pharmacol.* **122(1),** 149–157.
92. Kubo, S., Takahashi, H.K., Takei, M., Iwagaki, H., Yoshino, T., Tanaka, N., Mori, S., and Nishibori, M. (2004) E-prostanoid (EP)2/EP4 receptor-dependent maturation of human monocyte-derived dendritic cells and induction of helper T2 polarization. *J. Pharmacol. Exp. Ther.* **309(3),** 1213–1220.
93. Morichika, T., Takahashi, H.K., Iwagaki, H., Yagi, T., Saito, S., Kubo, S., Yoshino, T., Akagi, T., Mori, S., Nishibori, M., and Tanaka, N. (2003) Effect of prostaglandin E2 on intercellular adhesion molecule-1 and B7 expression in mixed lymphocyte reaction. *Transplantation* **75(12),** 2100–2105.
94. Hinz, B., Brune, K., and Pahl, A. (2000) Cyclooxygenase-2 expression in lipopolysaccharide-stimulated human monocytes is modulated by cyclic AMP, prostaglandin E(2), and nonsteroidal anti-inflammatory drugs. *Biochem. Biophys. Res. Commun.* **278(3),** 790–796.
95. Iwasaki, K., Noguchi, K., Endo, H., Kondo, H., and Ishikawa, I. (2003) Prostaglandin E2 downregulates interleukin-12 production through EP4 receptors in human monocytes stimulated with lipopolysaccharide from Actinobacillus actinomycetemcomitans and interferon-gamma. *Oral Microbiol. Immunol.* **18(3),** 150–155.
96. Zeng, L., An, S., and Goetzl, E.J. (1995) Independent down-regulation of EP2 and EP3 subtypes of the prostaglandin

E2 receptors on U937 human monocytic cells. *Immunology* **86(4),** 620–628.

97. Hoper, M.M., Voelkel, N.F., Bates, T.O., Allard, J.D., Horan, M., Shepherd, D., and Tuder, R.M. (1997) Prostaglandins induce vascular endothelial growth factor in a human monocytic cell line and rat lungs via cAMP. *Am. J. Respir. Cell Mol. Biol.* **17(6)**, 748–756.
98. Wagner, R.S., Halushka, P.V., and Cook, J.A. (1996) Activation of thromboxane A2 receptors alters lipopolysaccharide-induced adherence of THP-1 cells. *Shock* **5(1),** 41–46.
99. Takayama, K., Garcia-Cardena, G., Sukhova, G.K., Comander, J., Gimbrone, M.A., Jr., and Libby, P. (2002) Prostaglandin E2 suppresses chemokine production in human macrophages through the EP4 receptor. *J. Biol. Chem.* **277(46),** 44147–44154.
100. Luft, T., Jefford, M., Luetjens, P., Toy, T., Hochrein, H., Masterman, K.A., Maliszewski, C., Shortman, K., Cebon, J., and Maraskovsky, E. (2002) Functionally distinct dendritic cell (DC) populations induced by physiologic stimuli: prostaglandin E(2) regulates the migratory capacity of specific DC subsets. *Blood* **100(4),** b1362–1372.
101. Mori, K., Tanaka, I., Kotani, M., Miyaoka, F., Sando, T., Muro, S., Sasaki, Y., Nakagawa, O., Ogawa, Y., Usui, T., Ozaki, S., Ichikawa, A., Narumiya, S., and Nakao, K. (1996) Gene expression of the human prostaglandin E receptor EP4 subtype: differential regulation in monocytoid and lymphoid lineage cells by phorbol ester. *J. Mol. Med.* **74(6),** 333–336.
102. Zeng, L., An, S., and Goetzl, E.J. (1996) Regulation of expression of matrix metalloproteinase-9 in early human T cells of the HSB.2 cultured line by the EP3 subtype of prostaglandin E2 receptor. *J. Biol. Chem.* **271(44),** 27744–27750.
103. Cosme, R., Lublin, D., Takafuji, V., Lynch, K., and Roche, J.K. (2000) Prostanoids in human colonic mucosa: effects of inflammation on PGE(2) receptor expression. *Hum. Immunol.* **61(7),** 684–696.
104. Zeng, L., An, S., and Goetzl, E.J. (1998) EP4/EP2 receptor-specific prostaglandin E2 regulation of interleukin-6 generation by human HSB.2 early T cells. *J. Pharmacol. Exp. Ther.* **286(3)**, 1420–1426.
105. Goetzl, E.J., An, S., and Zeng, L. (1995) Specific suppression by prostaglandin E2 of activation-induced apoptosis of human CD4+CD8+ T lymphoblasts. *J. Immunol.* **154(3),** 1041–1047.
106. Clapp, L.H., Finney, P., Turcato, S., Tran, S., Rubin, L.J., and Tinker, A. (2002) Differential effects of stable prostacyclin analogs on smooth muscle proliferation and cyclic AMP generation in human pulmonary artery. *Am. J. Respir. Cell Mol. Biol.* **26(2),** 194–201.
107. Meyer-Kirchrath, J., Debey, S., Glandorff, C., Kirchrath, L., and Schror, K. (2004) Gene expression profile of the Gs-coupled prostacyclin receptor in human vascular smooth muscle cells. *Biochem. Pharmacol.* **67(4),** 757–765.
108. Debey, S., Kirchrath, L., Schror, K., and Meyer-Kirchrath, J. (2003) Iloprost down-regulates the expression of the growth regulatory gene Cyr61 in human vascular smooth muscle cells. *Eur. J. Pharmacol.* **474(2–3),** 161–164.
109. Walton, L.J., Franklin, I.J., Bayston, T., Brown, L.C., Greenhalgh, R.M., Taylor, G.W., and Powell, J.T. (1999) Inhibition of prostaglandin E2 synthesis in abdominal aortic aneurysms: implications for smooth muscle cell viability, inflammatory processes, and the expansion of abdominal aortic aneurysms. *Circulation* **100(1),** 48–54.
110. Proudfoot, D., Fitzsimmons, C., Torzewski, J., and Bowyer, D.E. (1999) Inhibition of human arterial smooth muscle cell growth by human monocyte/macrophages: a co-culture study. *Atherosclerosis* **145(1),** 157–165.
111. Goncharova, E.A., Billington, C.K., Irani, C., Vorotnikov, A.V., Tkachuk, V.A., Penn, R.B., Krymskaya, V.P., and Panettieri, R.A., Jr. (2003) Cyclic AMP-mobilizing agents and glucocorticoids modulate human smooth muscle cell migration. *Am. J. Respir. Cell Mol. Biol.* **29(1),** 19–27.
112. Kuwano, T., Nakao, S., Yamamoto, H., Tsuneyoshi, M., Yamamoto, T., Kuwano, M., and Ono, M. (2004) Cyclooxygenase 2 is a key enzyme for inflammatory cytokine-induced angiogenesis. *FASEB J.* **18(2),** 300–310.
113. Leahy, K.M., Ornberg, R.L., Wang, Y., Zweifel, B.S., Koki, A.T., and Masferrer, J.L. (2002) Cyclooxygenase-2 inhibition by celecoxib reduces proliferation and induces apoptosis in angiogenic endothelial cells in vivo. *Cancer Res.* **62(3),** 625–631.
114. Jones, M.K., Wang, H., Peskar, B.M., Levin, E., Itani, R.M., Sarfeh, I.J., and Tarnawski, A.S. (1999) Inhibition of angiogenesis by nonsteroidal anti-inflammatory drugs: insight into mechanisms and implications for cancer growth and ulcer healing. *Nat. Med.* **5(12),** 1418–1423.
115. Sennlaub, F., Valamanesh, F., Vazquez-Tello, A., El-Asrar, A.M., Checchin, D., Brault, S., Gobeil, F., Beauchamp, M.H., Mwaikambo, B., Courtois, Y., Geboes, K., Varma, D.R., Lachapelle, P., Ong, H., Behar-Cohen, F., and Chemtob, S. (2003) Cyclooxygenase-2 in human and experimental ischemic proliferative retinopathy. *Circulation* **108(2),** 198–204.
116. Chi-Man Tang, T., Tung-Ping Poon, R., and Fan, S.T. (2005) The significance of cyclooxygenase-2 expression in human hepatocellular carcinoma. *Biomed. Pharmacother.* **59(Suppl 2),** S311–316.
117. Mobius, C., Stein, H.J., Spiess, C., Becker, I., Feith, M., Theisen, J., Gais, P., Jutting, U., and Siewert, J.R. (2005) COX2 expression, angiogenesis, proliferation and survival in Barrett's cancer. *Eur. J. Surg. Oncol.* **31(7),** 755–759.
118. Marrogi, A.J., Travis, W.D., Welsh, J.A., Khan, M.A., Rahim, H., Tazelaar, H., Pairolero, P., Trastek, V., Jett, J., Caporaso, N.E., Liotta, L.A., and Harris, C.C. (2000) Nitric oxide synthase, cyclooxygenase 2, and vascular endothelial growth factor in the angiogenesis of non-small cell lung carcinoma. *Clin. Cancer Res.* **6(12),** 4739–4744.
119. Tang, T.C., Poon, R.T., Lau, C.P., Xie, D., and Fan, S.T. (2005) Tumor cyclooxygenase-2 levels correlate with tumor invasiveness in human hepatocellular carcinoma. *World J. Gastroenterol.* **11(13),** 1896–1902.
120. Amano, H., Hayashi, I., Endo, H., Kitasato, H., Yamashina, S., Maruyama, T., Kobayashi, M., Satoh, K., Narita, M.,

Sugimoto, Y., Murata, T., Yoshimura, H., Narumiya, S., and Majima, M. (2003) Host prostaglandin E(2)-EP3 signaling regulates tumor-associated angiogenesis and tumor growth. *J. Exp. Med.* **197(2),** 221–232.
121. Coleman, R.A., Smith, W.L., and Narumiya, S. (1994) International Union of Pharmacology classification of prostanoid receptors: properties, distribution, and structure of the receptors and their subtypes. *Pharmacol. Rev.* **46(2),** 205–229.
122. Abramovitz, M., Adam, M., Boie, Y., Carriere, M., Denis, D., Godbout, C., Lamontagne, S., Rochette, C., Sawyer, N., Tremblay, N.M., Belley, M., Gallant, M., Dufresne, C., Gareau, Y., Ruel, R., Juteau, H., Labelle, M., Ouimet, N., and Metters, K.M. (2000) The utilization of recombinant prostanoid receptors to determine the affinities and selectivities of prostaglandins and related analogs. *Biochim. Biophys. Acta* **1483(2),** 285–293.
123. Wilson, R.J. and Giles, H. (2005) Piglet saphenous vein contains multiple relaxatory prostanoid receptors: evidence for EP4, EP2, DP and IP receptor subtypes. *Br. J. Pharmacol.* **144(3),** 405–415.
124. Wilson, R.J., Rhodes, S.A., Wood, R.L., Shield, V.J., Noel, L.S., Gray, D.W., and Giles, H. (2004) Functional pharmacology of human prostanoid EP2 and EP4 receptors. *Eur. J. Pharmacol.* **501(1–3),** 49–58.
125. Angulo, J., Cuevas, P., La Fuente, J.M., Pomerol, J.M., Ruiz-Castane, E., Puigvert, A., Gabancho, S., Fernandez, A., Ney, P., and Saenz De Tejada, I. (2002) Regulation of human penile smooth muscle tone by prostanoid receptors. *Br. J. Pharmacol.* **136(1),** 23–30.
126. Baxter, G.S., Clayton, J.K., Coleman, R.A., Marshall, K., Sangha, R., and Senior, J. (1995) Characterization of the prostanoid receptors mediating constriction and relaxation of human isolated uterine artery. *Br. J. Pharmacol.* **116(1),** 1692–1696.
127. Davis, R.J., Murdoch, C.E., Ali, M., Purbrick, S., Ravid, R., Baxter, G.S., Tilford, N., Sheldrick, R.L., Clark, K.L., and Coleman, R.A. (2004) EP4 prostanoid receptor-mediated vasodilatation of human middle cerebral arteries. *Br. J. Pharmacol.* **141(4),** 580–585.
128. Qian, Y.M., Jones, R.L., Chan, K.M., Stock, A.I., and Ho, J.K. (1994) Potent contractile actions of prostanoid EP3-receptor agonists on human isolated pulmonary artery. *Br. J. Pharmacol.* **113(2),** 369–374.
129. Walch, L., de Montpreville, V., Brink, C., and Norel, X. (2001) Prostanoid EP(1)- and TP-receptors involved in the contraction of human pulmonary veins. *Br. J. Pharmacol.* **134(8),** 1671–1678.
130. Walch, L., Labat, C., Gascard, J.P., de Montpreville, V., Brink, C., and Norel, X. (1999) Prostanoid receptors involved in the relaxation of human pulmonary vessels. *Br. J. Pharmacol.* **126(4),** 859–866.
131. Martin-Ventura, J.L., Blanco-Colio, L.M., Gomez-Hernandez, A., Munoz-Garcia, B., Vega, M., Serrano, J., Ortega, L., Hernandez, G., Tunon, J., and Egido, J. (2005) Intensive treatment with atorvastatin reduces inflammation in mononuclear cells and human atherosclerotic lesions in one month. *Stroke* **36(8)**, 1796–1800.
132. Jouve, R., Rolland, P.H., Delboy, C., and Mercier, C. (1984) Thromboxane B2, 6-keto-PGF1 alpha, PGE2, PGF2 alpha, and PGA1 plasma levels in arteriosclerosis obliterans: relationship to clinical manifestations, risk factors, and arterial pathoanatomy. *Am. Heart J.* **107(1),** 45–52.
133. Parsons, A.A. and Whalley, E.T. (1989) Effects of prostanoids on human and rabbit basilar arteries precontracted in vitro. *Cephalalgia* **9(3),** 165–171.
134. Uski, T.K., Andersson, K.E., Brandt, L., and Ljunggren, B. (1984) Characterization of the prostanoid receptors and of the contractile effects of prostaglandin F2 alpha in human pial arteries. *Acta Physiol. Scand.* **121(4),** 369–378.
135. Hempelmann, R.G., Barth, H.L., Mehdorn, H.M., Pradel, R.H., and Ziegler, A. (1995) Effects of potassium channel openers in isolated human cerebral arteries. *Neurosurgery* **37(6),** 1146–1153.
136. Spada, C.S., Nieves, A.L., and Woodward, D.F. (2002) Vascular activities of prostaglandins and selective prostanoid receptor agonists in human retinal microvessels. *Exp. Eye Res.* **75(2),** 155–163.
137. Ohlstein, E.H., Kopia, G.A., Zeid, R.L., Valocik, R.W., Horohonich, S., Hieble, J.P., and Wasserman, M.A. (1988) Effects of the thromboxane receptor antagonist SK&F 88046 in the canine, monkey and human coronary vasculature. *Prostaglandins* **36(1),** 69–84.
138. Merritt, J.E., Brown, A.M., Bund, S., Cooper, D.G., Egan, J.W., Hallam, T.J., Heagerty, A.M., Hickey, D.M., Kaumann, A.J., Keen, M., et al. (1991) Primate vascular responses to octimibate, a non-prostanoid agonist at the prostacyclin receptor. *Br. J. Pharmacol.* **102(1)**, 260–266.
139. Cocks, T.M., Kemp, B.K., Pruneau, D., and Angus, J.A. (1993) Comparison of contractile responses to 5-hydroxytryptamine and sumatriptan in human isolated coronary artery: synergy with the thromboxane A2-receptor agonist, U46619. *Br. J. Pharmacol.* **110(1),** 360–368.
140. Champion, H.C., Bivalacqua, T.J., Pierce, R.L., Murphy, W.A., Coy, D.H., Hyman, A.L., and Kadowitz, P.J. (2003) Responses to human CGRP, ADM, and PAMP in human thymic arteries. *Am. J. Physiol. Regul. Integr. Comp. Physiol.* **284(2),** R531–537.
141. Maddox, Y., Cunard, C.M., Shapiro, R., Kawaguchi, A., Goldman, M., and Lower, R.R. (1985) *A Comparison of the Contractile Responses of Rodent and Human Pulmonary Vascular Segments to Eicosanoids.*: Elsevier.
142. Haye-Legrand, I., Bourdillat, B., Labat, C., Cerrina, J., Norel, X., Benveniste, J., and Brink, C. (1987) Relaxation of isolated human pulmonary muscle preparations with prostacyclin (PGI2) and its analogs. *Prostaglandins* **33(6),** 845–854.
143. Norel, X., Labat, C., Gardiner, P.J., and Brink, C. (1991) Inhibitory effects of BAY u3405 on prostanoid-induced contractions in human isolated bronchial and pulmonary arterial muscle preparations. *Br. J. Pharmacol.* **104(3),** 591–595.

144. Kamper, A.M., Paul, L.C., and Blauw, G.J. (2002) Prostaglandins are involved in acetylcholine- and 5-hydroxytryptamine-induced, nitric oxide-mediated vasodilatation in human forearm. *J. Cardiovasc. Pharmacol.* **40(6),** 922–929.
145. Arner, M. and Hogestatt, E.D. (1991) Endothelium-dependent relaxation and effects of prostacyclin, endothelin and platelet-activating factor in human hand veins and arteries. *Acta Physiol. Scand.* **142(2),** 165–172.
146. Arner, M., Hogestatt, E.D., and Uski, T.K. (1991) Characterization of contraction-mediating prostanoid receptors in human hand veins: effects of the thromboxane receptor antagonists BM13,505 and AH23848. *Acta Physiol. Scand.* **141(1),** 79–86.
147. Bjoro, K. and Stray-Pedersen, S. (1986) Effects of vasoactive autacoids on different segments of human umbilicoplacental vessels. *Gynecol. Obstet. Invest.* **22(1),** 1–6.
148. Boura, A.L., Gude, N.M., King, R.G., Mak, K.K., and Walters, W.A. (1986) Characterization of thromboxane A2 receptors in the human fetal placental vessels and umbilical vein. *Clin. Exp. Pharmacol. Physiol.* **13(1),** 83–86.
149. Boersma, J.I., Janzen, K.M., Oliveira, L., and Crankshaw, D.J. (1999) Characterization of excitatory prostanoid receptors in the human umbilical artery in vitro. *Br. J. Pharmacol.* **128(7),** 1505–1512.
150. Daray, F.M., Minvielle, A.I., Puppo, S., and Rothlin, R.P. (2003) Pharmacological characterization of prostanoid receptors mediating vasoconstriction in human umbilical vein. *Br. J. Pharmacol.* **139(8),** 1409–1416.
151. Cracowski, J.L., Stanke-Labesque, F., Sessa, C., Hunt, M., Chavanon, O., Devillier, P., and Bessard, G. (1999) Functional comparison of the human isolated femoral artery, internal mammary artery, gastroepiploic artery, and saphenous vein. *Can. J. Physiol. Pharmacol.* **77(10),** 770–776.
152. Furci, L., Fitzgerald, D.J., and Fitzgerald, G.A. (1991) Heterogeneity of prostaglandin H2/thromboxane A2 receptors: distinct subtypes mediate vascular smooth muscle contraction and platelet aggregation. *J. Pharmacol. Exp. Ther.* **258(1),** 74–81.
153. McLaughlin, V.V., Gaine, S.P., Barst, R.J., Oudiz, R.J., Bourge, R.C., Frost, A., Robbins, I.M., Tapson, V.F., McGoon, M.D., Badesch, D.B., Sigman, J., Roscigno, R., Blackburn, S.D., Arneson, C., Rubin, L.J., and Rich, S. (2003) Efficacy and safety of treprostinil: an epoprostenol analog for primary pulmonary hypertension. *J. Cardiovasc. Pharmacol.* **41(2),** 293–299.
154. Sussmann, M., Sarbia, M., Meyer-Kirchrath, J., Nusing, R.M., Schror, K., and Fischer, J.W. (2004) Induction of hyaluronic acid synthase 2 (HAS2) in human vascular smooth muscle cells by vasodilatory prostaglandins. *Circ. Res.* **94(5),** 592–600.
155. Cipollone, F., Fazia, M.L., Iezzi, A., Cuccurullo, C., De Cesare, D., Ucchino, S., Spigonardo, F., Marchetti, A., Buttitta, F., Paloscia, L., Mascellanti, M., Cuccurullo, F., and Mezzetti, A. (2005) Association between prostaglandin E receptor subtype EP4 overexpression and unstable phenotype in atherosclerotic plaques in human. *Arterioscler. Thromb. Vasc. Biol.* **25(9),** 1925–1931.
156. Inoue, H., Takamori, M., Shimoyama, Y., Ishibashi, H., Yamamoto, S., and Koshihara, Y. (2002) Regulation by PGE2 of the production of interleukin-6, macrophage colony stimulating factor, and vascular endothelial growth factor in human synovial fibroblasts. *Br. J. Pharmacol.* **136(2),** 287–295.
157. Schmitz, T., Leroy, M.J., Dallot, E., Breuiller-Fouche, M., Ferre, F., and Cabrol, D. (2003) Interleukin-1beta induces glycosaminoglycan synthesis via the prostaglandin E2 pathway in cultured human cervical fibroblasts. *Mol. Hum. Reprod.* **9(1),** 1–8.
158. Haveman, J.W., van den Berg, A.P., Verhoeven, E.L., Nijsten, M.W., van den Dungen, J.J., The, H.T., and Zwaveling, J.H. (2006) HLA-DR expression on monocytes and systemic inflammation in patients with ruptured abdominal aortic aneurysms. *Crit. Care* **10(4),** R119.
159. Gudmundsson, G., Matthiasson, S.E., Arason, H., Johannsson, H., Runarsson, F., Bjarnason, H., Helgadottir, K., Thorisdottir, S., Ingadottir, G., Lindpaintner, K., Sainz, J., Gudnason, V., Frigge, M.L., Kong, A., Gulcher, J.R., and Stefansson, K. (2002) Localization of a gene for peripheral arterial occlusive disease to chromosome 1p31. *Am. J. Hum. Genet.* **70(3),** 586–592.
160. Norel, X., de Montpreville, V., and Brink, C. (2004) Vasoconstriction induced by activation of EP1 and EP3 receptors in human lung: effects of ONO-AE-248, ONO-DI-004, ONO-8711 or ONO-8713. *Prostaglandins Other Lipid Mediat.* **74(1–4),** 101–112.
161. Matsuoka, T. and Narumiya, S. (2007) Prostaglandin receptor signaling in disease. *TheScientificWorldJOURNAL* **7**, 1329-1347.
